# Mapping medication adherence interventions across Europe: Expert perspectives from 35 countries

**DOI:** 10.1016/j.puhip.2026.100850

**Published:** 2026-09-09

**Authors:** S. Mucherino, E. Aarnio, M. Qvarnström, G. Hafez, M. Kamusheva, I. Potočnjak, I. Trečiokiene, J. Mihajlović, M. Ekenberg, J.F.M. van Boven, F. Leiva-Fernandez, European Network to Advance Best Practices and Technology on Medication AdherencE (ENABLE)

**Affiliations:** a Department of Pharmacy, University of Naples Federico II, Naples, NA, Italy; b Center of Pharmacoeconomics and Drug Utilization Research (CIRFF), University of Naples Federico II, Italy; c School of Pharmacy, University of Eastern Finland, Kuopio, Finland; d Department of Pharmacy, Faculty of Pharmacy, Uppsala University, Uppsala, Sweden; e Department of Pharmacology, Faculty of Pharmacy, Altinbas University, Istanbul, Turkey; f Department of Medical Pharmacology, Koç University School of Medicine, Istanbul, Turkey; g Faculty of Pharmacy, Medical University of Sofia, Sofia, Bulgaria; h Sestre Milosrdnice University Hospital Center; School of Medicine Catholic University of Croatia, Zagreb, Croatia; i Faculty of Medicine, Vilnius University, Vilnius, Lithuania; j University of Groningen, Department of Health Sciences, University Medical Center Groningen, Groningen, Netherlands; k Mihajlović Health Analytics, Novi Sad, Serbia; l University of Novi Sad, Medical Faculty, Novi Sad, Serbia; m Department of Clinical Pharmacy and Pharmacology, Medication Adherence Expertise Center of the Northern Netherlands (MAECON), University Medical Center Groningen, University of Groningen, Groningen, Netherlands; n Málaga-Valle Del Guadalhorce Health District-Andalusian Health Service-IBIMA-BIONAND Platform University of Malaga, Malaga, Spain

**Keywords:** Medication adherence, Adherence interventions, Healthcare professionals, Medication adherence experts

## Abstract

**Objectives:**

Medication adherence (MA) is a key challenge in healthcare. Literature about the real-world implementation of MA interventions across Europe remains poorly understood. This study aims to map MA interventions reported by experts across Europe.

**Study design:**

This cross-sectional study used a self-administered online survey targeting MA experts from 39 European countries. Experts were defined as professionals with clinical, policy, or research expertise in МА.

**Methods:**

Open-ended responses on expert-reported MA interventions were analysed using the framework method. The resulting categories were quantified at respondent level and described overall and by European region. Regional differences in the proportions of respondents reporting each intervention category were explored using exact tests, with adjustment for multiple comparisons.

**Results:**

A total of 140 MA experts from 35 European countries participated. Communication and education (83.6%) were the most frequently reported interventions, followed by follow-up and monitoring (53.6%), technological solutions (32.1%), and collaborative and multidisciplinary approaches (30.0%). Exploratory analyses identified regional differences in the reporting of collaborative and multidisciplinary approaches and technological solutions. Direct patient communication (35.0%) was the intervention most frequently perceived as successful by respondents and technological solutions were less frequently perceived as successful (12.9%).

**Conclusions:**

This study provides an expert-reported overview of MA interventions across European settings. Communication and education were the most frequently reported approaches, whereas technological and multidisciplinary interventions were reported less often. These findings support the need for further evaluation and more systematic integration of MA interventions across diverse European healthcare settings.

## 1 Introduction

The implementation of interventions to enhance medication adherence (MA) is essential in healthcare, as MA involves the intricate interplay of patient behaviour, healthcare practices, and systemic factors. To date, several global studies have examined determinants of MA; however, the effectiveness, cost-effectiveness and real-world application of different intervention strategies remain an area of ongoing research [1,2]. Reported MA interventions include educational, behavioural, technological, and collaborative strategies, often used in combination [[3], [4], [5], [6], [7], [8], [9], [10], [11]]. However, the success of these interventions depends on the specific challenges and contexts within diverse healthcare systems. In this scenario, recognizing the need for a concerted effort to address MA across Europe, the European Cooperation in Science and Technology (COST) initiated the “European Network to Advance Best practices & technoLogy on medication adherencE” (ENABLE) project [12]. This sub-study explores how MA experts believe MA interventions are implemented in their country. Specifically, the study mission was to identify key insights and best practices that can guide the development of more effective MA interventions, by analysing expert experiences across Europe. This research expands current knowledge and provides recommendations to improve MA interventions in the European healthcare context. Since new approaches for providing healthcare and follow-up of patients were implemented during the COVID-19 pandemic, this research also explored the adaptation of MA-enhancing interventions during the pandemic. The present study explored experts’ reports of MA interventions across European healthcare settings. We aimed to: (i) identify the professional groups involved in MA management; (ii) map the interventions reported as available when non-adherence was identified; (iii) identify the interventions perceived by experts as most successful; and (iv) describe adaptations reported during the COVID-19 pandemic.

## 2 Methods

### 2.1 Study design

The study design consisted of a cross-sectional study carried out using a self-administered online survey of MA experts from European countries. The survey included open-ended questions designed to collect expert-reported information on MA interventions. A sequential analytical approach was used. First, open-ended responses were analysed using the framework method and grouped into analytical categories and subcategories. The resulting categories were then quantified at the respondent level and described overall and by the three European regions: Western, Central and Eastern [13]. The detailed study protocol has been registered in the Open Science Framework (OSF) [14]. This study was reported according to the CHERRIES checklist for online surveys [15]. The survey was first piloted among 12 MA experts from 5 European countries from the COST ENABLE network (Bulgaria, Croatia, Spain, Sweden, Turkey) (see Supplementary File 1). The pilot study aimed to validate the questions and to refine the study protocol and the information to be used to contact the experts (cover letter, invitation and reminder emails). Responses to the pilot study were not included in the final study data but were used to make an initial coding list for qualitative analysis (Supplementary File 1, Tables S1–S13). Data were collected through the same expert survey previously used to investigate barriers and unmet educational needs in MA management [16]. The previous publication reported findings from different survey domains. The present study analyses Questions 1, 5, 6, and 10, focusing on professional roles, interventions offered to address non-adherence, interventions perceived as most successful, and initiatives reported during the COVID-19 pandemic.

### 2.2 Eligibility criteria and study population

The online survey was designed for MA experts from healthcare, academic and governmental institutions, and patient associations. The survey was then sent to these experts operating in 39 European countries: Albania, Austria, Belgium, Bosnia and Herzegovina, Bulgaria, Croatia, Cyprus, Czech Republic, Denmark, Estonia, Finland, France, Germany, Greece, Hungary, Iceland, Ireland, Israel, Italy, Latvia, Lithuania, Luxembourg, Malta, Moldova, Montenegro, Netherlands, North Macedonia, Norway, Poland, Portugal, Romania, Serbia, Slovakia, Slovenia, Spain, Sweden, Switzerland, Turkey, and the United Kingdom. A purposive, network-based sampling strategy was used: COST ENABLE representatives from each participating country were asked to identify experts based on their clinical, policy, and/or research expertise in MA and their knowledge of national practices. The recruitment strategy sought to include experts from different professional and organisational backgrounds, including healthcare, academia or research, policymaking, and patient organisations. The intended recruitment target was at least five experts per country, with up to eight experts included when additional eligible participants were identified. Participation was voluntary and anonymous, and all respondents provided online informed consent.

### 2.3 Survey administration

The online survey was open from April to June 2021. The survey was created using the online Webropol 3.0 survey and reporting tool (https://webropol.com/) and was available only in English. Respondents had the option to answer in their native language. The complete survey is provided in Supplementary File 1 Table S14.

### 2.4 Outcomes

The outcomes concerned: (i) professional groups involved in MA management (question #1); (ii) interventions offered when non-adherence was identified (question #5); (iii) interventions perceived as most successful (question #6); and (iv) MA initiatives during the COVID-19 pandemic (question #10). The corresponding survey questions are provided in Supplementary File 1, Table S14.

### 2.5 Qualitative analysis

Open-ended responses were analysed using the framework method [17]. This method was used as the survey included focused open-ended questions asking experts to describe concrete MA interventions within their national contexts. An initial coding framework was developed from the pilot study. The survey questions were divided among pairs of researchers, with each pair analysing two or three questions. Within each pair, the two researchers independently reviewed all responses to the assigned questions and assigned one or more codes to each response. Each researcher completed the initial coding without access to the other researcher's coding decisions. New codes were added when relevant concepts were not represented in the initial framework. After independent coding, the two researchers compared their coding and resolved disagreements through discussion. The agreed codes and any unresolved issues were then reviewed by the full research team. Final decisions were reached by consensus. The framework was therefore refined iteratively using both codes derived from the pilot study and codes emerging from the full dataset. For Questions 5 and 6, the final codes were organized into five overarching categories: communication and education; follow-up and monitoring; collaborative and multidisciplinary approaches; technological solutions; and uncertain or varied approaches. Each category included more specific subcategories. Separate coding frameworks were developed for the professional groups involved in MA management and for COVID-19-related initiatives.

### 2.6 Quantitative analysis and regional comparisons

After qualitative analysis, the agreed codes were converted into respondent-level categorical variables, with each open-ended response as the unit of analysis. Responses could receive multiple codes, but repeated mentions of the same code were counted once. Respondents were counted once per category when at least one corresponding subcategory was identified; thus, categories were not mutually exclusive. Frequencies and percentages were calculated for each code and category using the total sample (n = 140) or the respective regional samples as denominators. The same categories informed the qualitative framework and quantitative results. Countries were grouped according to the Global Burden of Disease regional classification [13]. Regional comparisons were descriptive and exploratory; population- or country-level representativeness was not assumed because respondent and country counts differed across regions. Analyses treated respondents as independent and did not account for clustering within countries. Regional differences were explored separately for intervention categories reported by experts (Question #5) and those perceived as successful (Question #6). The association between macro-category reporting and European region was assessed using the Fisher–Freeman–Halton exact test for 3 × 2 contingency tables. P-values were adjusted separately for each set of outcomes using the Benjamini–Hochberg procedure. Cramér's V with 95% confidence intervals, estimated using the noncentral chi-squared distribution, was calculated as a measure of association. A p-value of less than 0.05 was considered statistically significant. Analyses were performed using SPSS version 27 and R for statistical computing version 4.5.2.

## 3 Results

### 3.1 Study population characteristics

The final sample included 140 respondents from 35 countries, with the number of respondents ranging from one to eight per country. Because recruitment was coordinated independently by national COST ENABLE representatives, the total number of individuals invited was not centrally recorded. Country-specific numbers and percentages are reported in Supplementary File 2, Figs. S1 and S2. Of the 140 respondents, 63 (45.0%) were from Central, 61 (43.6%) were from Western and 16 (11.4%) were from Eastern European countries (Supplementary File 2: Figs. S1 and S2). The Eastern European group included respondents from four countries: Estonia, Latvia, Lithuania, and Turkey. Owing to the smaller size and limited country coverage of this group, regional findings are presented as descriptive patterns. Most MA experts represented a research/academic organization (52.9%), followed by hospital (27.9%), primary care (19.3%) and community or hospital pharmacy (17.1%) settings. Table 1 shows an overview of the professions involved in MA management across European countries. When asked which healthcare professionals were involved in supporting medication adherence, 90.7% of respondents mentioned physicians and clinical pharmacologists, 70.7% mentioned pharmacists, and 55.7% mentioned nurses.

**Table 1:** Professions involved in MA management in European countries.

Question #1. Which professions are involved in medication adherence in your country and what is their role in evaluating, monitoring, documenting and/or improving adherence?^a^	Europe (n = 140) N (%)	Western regions (n = 61, 43.6%) N (%)	Central regions (n = 63, 45.0%) N (%)	Eastern regions (n = 16, 11.4%) N (%)
**Physicians/Doctors: Primary care, family doctors (GPs) and medical specialists, clinical pharmacologists**	127 (90.7%)	54 (88.5%)	61 (96.8%)	12 (75.0%)
**Pharmacists (community and hospital, clinical pharmacists)**	99 (70.7%)	46 (75.4%)	44 (69.8%)	9 (56.3%)
**Nurses**	78 (55.7%)	44 (72.1%)	26 (41.3%)	8 (50.0%)
**Other (Medical technicians and physiotherapists, midwives, nursing aides, assistants, social services)**	14 (10.0%)	7 (11.5%)	6 (9.5%)	1 (6.3%)
**Psychologists**	10 (7.1%)	4 (6.6%)	6 (9.5%)	-
**National health insurance institutes**	7 (5.0%)	-	7 (11.1%)	-
**Researchers**	5 (3.6%)	3 (4.9%)	1 (1.6%)	1 (6.3%)
**Dentists**	3 (2.1%)	1 (1.6%)	2 (3.2%)	-
**Home Care Services**	3 (2.1%)	2 (3.3%)	1 (1.6%)	-

a Respondents could report more than one professional group, and each response could therefore be assigned to multiple professional categories.

### 3.2 Interventions to improve medication adherence

**Fig. 1:**
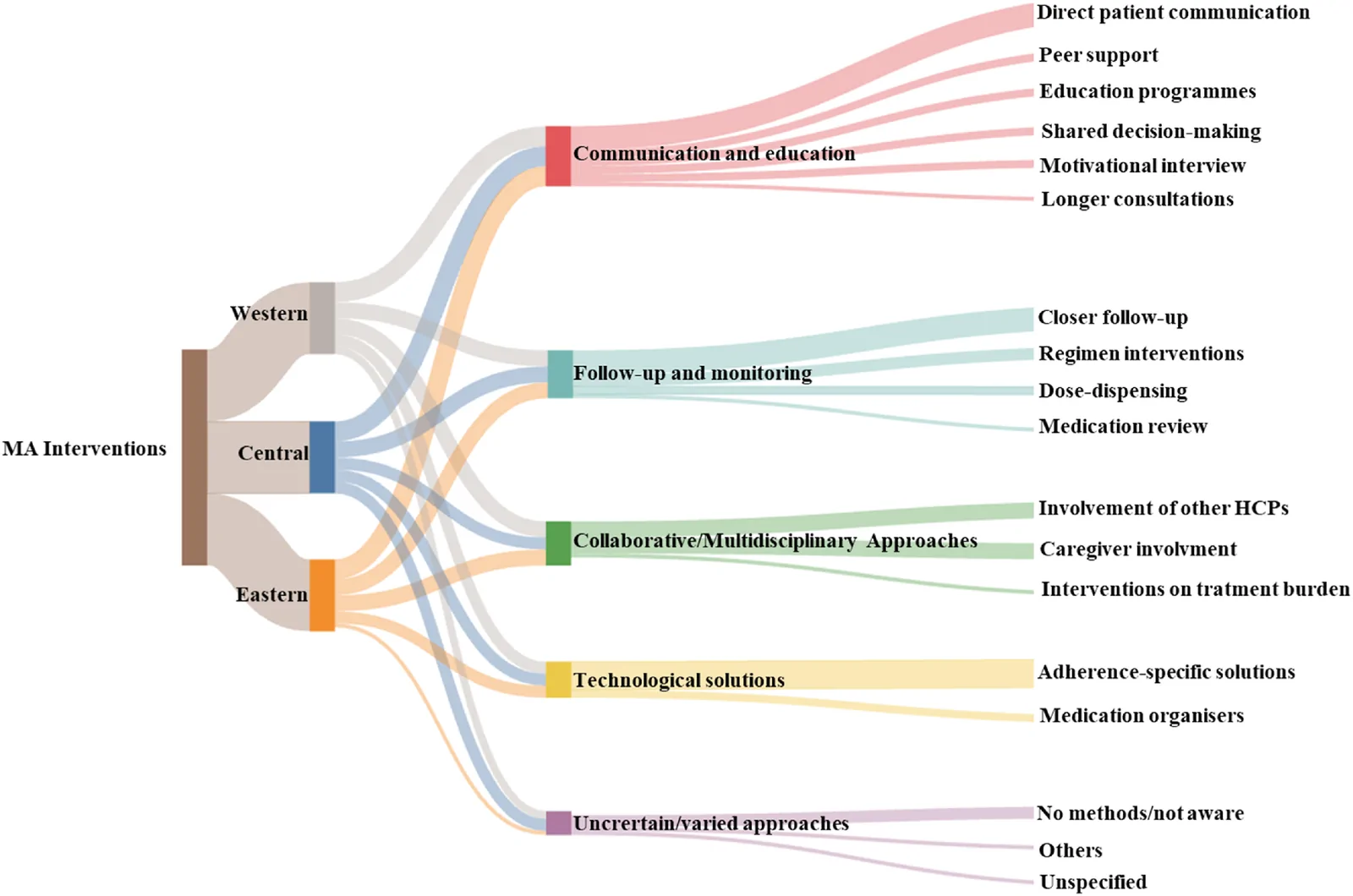
Available interventions to improve MA identified by MA experts across European regions (question #5). Footnotes: The Sankey diagram illustrates the distribution of medication adherence (MA) interventions indicated by experts from Western (n = 61), Central (n = 63), and Eastern (n = 16) European regions. Each segment represents a specific MA intervention category. Flow widths represent the proportion of respondents within each region reporting the corresponding intervention category. Percentages were calculated using the total number of respondents in each region as the denominator. Respondents could report more than one intervention category.

Communication and education were the most frequently reported intervention category among all three European regions' respondents (83.6% Western, 87.3% Central and 68.8% Eastern) (Fig. 1 and Supplementary File 2 Table S1). Specifically, direct patient communication, combined with appropriate patient education, was the most frequently reported intervention across all European respondents. Follow-up and monitoring were also frequently reported, with variations across Western (60.7%), Central (49.2%) and Eastern European respondents (43.8%). Exploratory analyses indicated overall regional differences in the reporting of collaborative and multidisciplinary approaches (BH-adjusted exact p = 0.046; Cramér's V = 0.242, 95% CI 0.041–0.396) and technological solutions (BH-adjusted exact p = 0.046; Cramér's V = 0.243, 95% CI 0.042–0.397). Both categories were reported more frequently by respondents from Western Europe.

### 3.3 Perceived success of adherence interventions

**Fig. 2:**
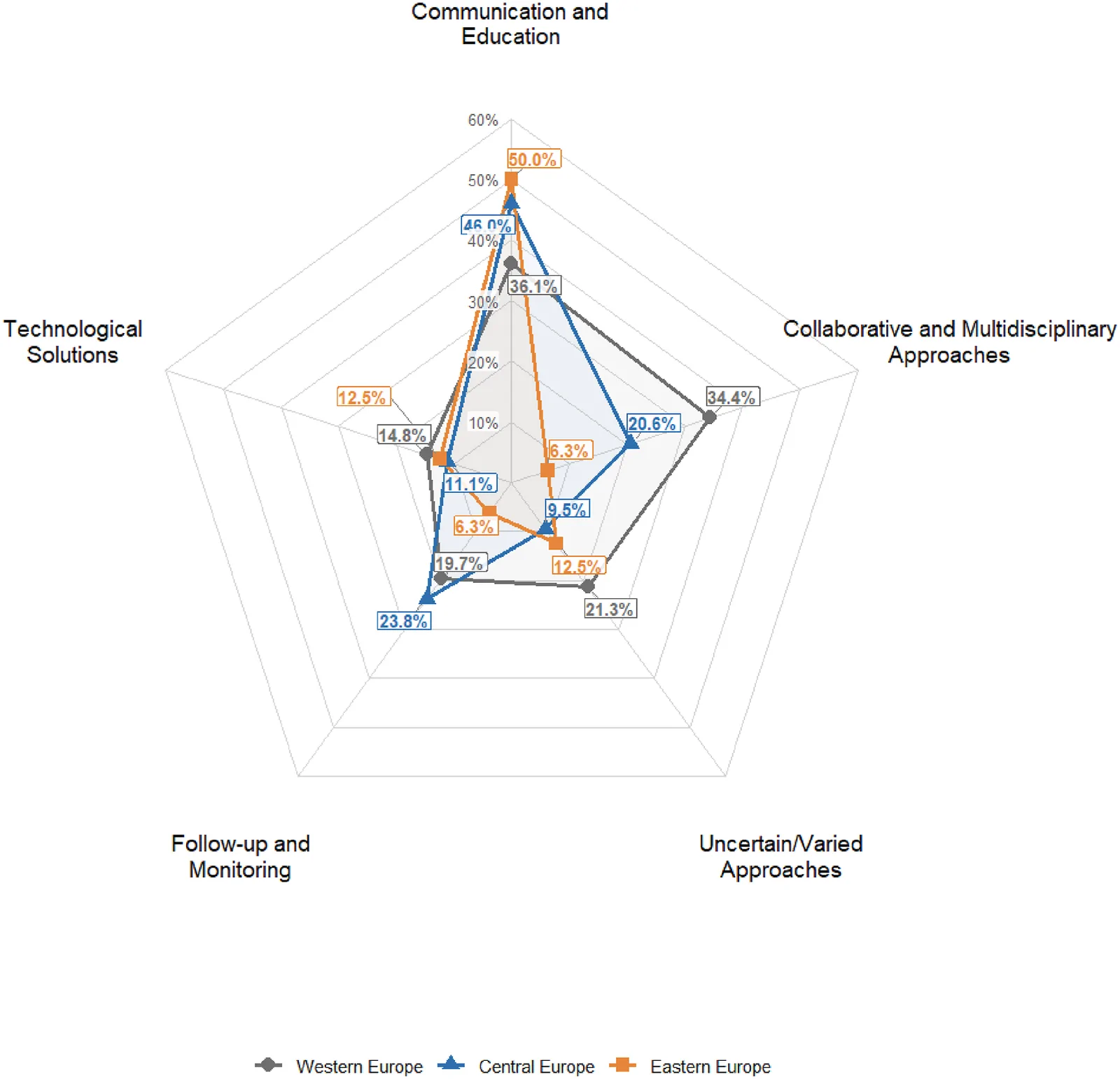
Medication adherence intervention categories perceived as successful by experts across European regions (question #6). Notes: Values represent the percentage of respondents within each region who identified at least one intervention within the corresponding macro-category as successful. Regional denominators were Western Europe, n = 61; Central Europe, n = 63; and Eastern Europe, n = 16. Respondents could contribute to more than one macro-category. Complete numerical results, including counts, adjusted exact p-values and effect sizes, are reported in Supplementary File 2, Table S2.

Table 2 summarizes the interventions perceived as successful by respondents. Direct patient communication, including motivational interviewing, shared decision-making, longer consultations, peer support and patient training, was the intervention most frequently perceived as successful overall (35%; Fig. 2 and Supplementary File 2: Table S2). At the macro-category level, communication and education was the category most frequently perceived as successful by respondents from Western, Central and Eastern Europe (36.1%, 46.0% and 50%, respectively). Descriptively, collaborative and multidisciplinary approaches were reported more frequently by Western European respondents (34.4%) than by Central (20.6%) and Eastern European respondents (6.3%). Follow-up and monitoring were reported by 19.7% of Western, 23.8% of Central and 6.3% of Eastern European respondents. However, none of the intervention macro-categories showed a statistically significant regional association after adjustment for multiple comparisons (all BH-adjusted exact p ≥ 0.209, see Supplementary File 2: Table S2).

**Table 2:** Analytical framework, categories, subcategories, and illustrative quotations for medication adherence interventions offered and perceived as successful from experts.

Categories & codes	Example quotes^a^ question #5 Medication adherence interventions offered	Example quotes^b^ question #6 Medication adherence interventions perceived as successful
**Communication and education**		
Direct patient communication (incl. Education, and patient-HCP relation)	*“Mainly, to assess through a clinical interview the obstacles that are motivating non-adherence and to seek solutions to it.”* *“They can only be consulted during a general visit.”*	*“The [explanation] to the patient and the patient understanding of seriousness of his/her disease and why it is so important to adhere the treatment.”* *“A closer doctor-patient relationship based on mutual trust.”*
Peer support (incl. Collaboration with patient associations)	*“referral to peer-support groups, patient empowerment societies …”* *“… participation in patient advocacy/cancer survival groups.”*	*“Training with expert [patients]”* *“For the professionals, their training.”*
Education programmes (incl. Self-care training)	*“Self-care training programmes”* *“… for some special diseases can send for specialized lessons.”*	*“[Self-care] training programmes”*
Shared decision-making	*“… patient involvement in decision-making on treatment”*	*“… (HCPs listen to the patient and seeking solutions together) …”*
Motivational interview	*“… perform motivational interviewing …”*	*“… motivational interviewing for unintentional [nonadherence]”*
Longer consultations	*“… longer consultations.”*	*“… consultation must last long …”*
**Follow-up and monitoring**		
Closer follow-up	*“more frequent visits to the doctor”*	*“… provide additional consultation or provide closer follow-up”*
Regimen interventions	*“Medication could also be modified (e.g. change dosage form of the medication, change to extended-release products, simplify patient's treatment).”*	*“Simplification of treatments”*
Dose-dispensing	*“… pharmacy dispensed doses …”*	*“Pharmacy dispensed doses”*
Medication review	*“… perform MUR”*	*“Medication use review by pharmacists …”*
**Technological solutions**		
Technological solutions specific for adherence (*e.g.* mobile apps, reminders)	*“… installing applications on mobile devices that will inform the patient not to miss the time to take the medicine …”* *“… use of mobile or other applications …”*	*“… using reminder tools and mobile applications …”*
Medication organizers	*“Use of pill organizers through pharmacies”*	*“Pillboxes …”*
**Collaborative and multidisciplinary approaches**		
Involvement of other HCPs (incl. home care)	*“Collaboration with GP and homecare staff”*	*“… being able to involve other actors such as pharmacologists and pharmacists is also very important to improve adherence …”*
Caregiver involvement (incl. education)	*“… involve someone from the patient's family to help …”*	*“cooperation with family caregiver”*
Other adherence interventions	*“… if the patient does not have sufficient financial resources, contact the Social Worker …”*	*“Development and implementation of organized PSP (patients supporting programmes)”* *“In my experience, a combination of interventions is often the best one …”*
**Uncertain/varied approaches**		
Other	*“develop a medication plan appropriate to their needs and beliefs”*	*“… medical call center service/online consultations”*
Unspecified adherence interventions	*“… be invited to participate in dedicated programs …”*	*“… depends on the individual needs of the patients.”*
Unaware	*“I don't know about programs”*	*“It is difficult to say how effective, in my opinion, any educational campaigns are …”*
Punishing measures	*“… the physicians can refuse his further examinations …”*	
**No methods available**	*“… there are no systematic adherence programs on national level ….”*	*“There is no best practice for that. There is a clear unmet medical need on this field”*

HCP: healthcare professional, MUR: Medicines Use Review.

a Coding of responses to question #5: If a patient is identified as being non-adherent, what interventions can health professionals offer to improve adherence (for example, give consultation, organize closer follow-up, refer to an adherence intervention/program, mobile applications)?.

b Coding of responses to question #6: What would you identify as the most successful adherence interventions in your country within the interventions you mentioned in your answer to question 5?.

### 3.4 Specific interventions to improve MA during the COVID-19 pandemic

**Fig. 3:**
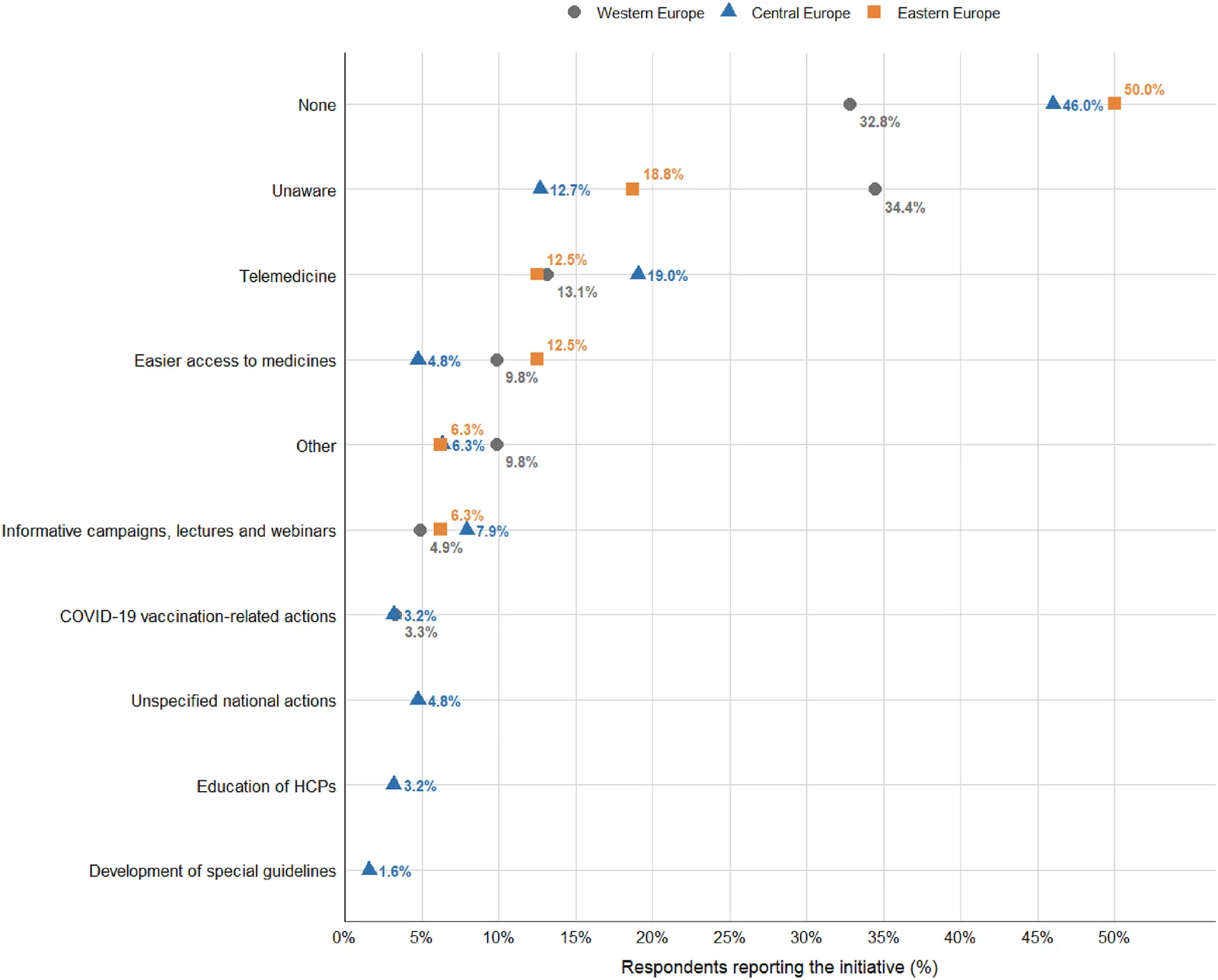
Medication adherence initiatives reported by experts during the COVID-19 pandemic across European regions (question #10). Notes: Values represent the percentage of respondents within each region reporting each COVID-19-related initiative. Values of 0% are not displayed. Regional denominators were Western Europe, n = 61; Central Europe, n = 63; and Eastern Europe, n = 16. Respondents could report more than one initiative. Findings are descriptive and reflect experts' retrospective reports. Complete numerical results are provided in Supplementary File 2, Table S3.

During the pandemic, 40.7% of experts reported no specific MA initiatives in their countries (Fig. 3 and Supplementary File 2 Table S3). This finding should be interpreted cautiously because responses were retrospective and depended on respondents’ awareness of national or local initiatives. Telemedicine, including e-prescriptions, was reported by 15.7% of respondents. Easier access to medicines, facilitated by services such as pharmacists and home delivery, was reported by 7.9% of respondents. Informative campaigns, lectures, and webinars were mentioned in 6.4% of responses. In this sample, respondents from Central Europe reported a wider range of initiatives, including vaccination-related actions and educational programmes for healthcare professionals.

## 4 Discussion

### 4.1 Main findings

MA experts most frequently reported communication with and education of patients as approaches that HCPs could use to address medication non-adherence. These were followed by closer follow-up, collaborative approaches, and technological solutions. Direct patient communication, including motivational interviewing and shared decision-making, was the intervention most frequently perceived as successful. Perceived success differed descriptively across European respondents. Indeed, respondents from Western Europe cited collaborative/multidisciplinary approaches, while those from Central Europe highlighted follow-up/monitoring. A key finding of this study is the central role of HCPs in MA management, especially physicians, pharmacists, and nurses, both at primary care and hospital level. This aligns with existing literature, emphasizing the importance of HCPs in tailoring interventions to patient-specific needs and addressing barriers to adherence [[18], [19], [20], [21], [22], [23], [24]]. However, the observed variations in the involvement of HCPs across regions highlight the need for tailored interventions that consider regional contexts and healthcare structures. Notably, technology-enabled adherence tools were reported less often and were less frequently perceived as successful. These approaches align with previously published literature, which has, however, identified them as only moderately effective in addressing MA challenges [4]. In fact, the limited uptake of technological solutions could reflect practical barriers, such as uncertain reimbursement, unclear integration with electronic health records/pharmacy systems, data privacy, variable digital literacy and access among older or multimorbid patients [25]. The higher prevalence of direct patient communication aligns with the patient-centred care model, emphasizing the significance of personalized interactions in promoting MA [[26], [27], [28], [29]], and could also explain why physicians, pharmacists and nurses specifically are highly involved in MA management. Moreover, HCP training for an effective communication with patients has been extensively studied and associated with improvements in the communication skills of HCPs [[30], [31], [32], [33]]. Improved communication combined with closer and more frequent follow-up visits with patients offers the opportunity to discuss aspects of the disease and treatment with the patient during the clinical encounter. This would help to achieve an adaptive understanding of the problem, which has been proven to be effective in enhancing MA [[34], [35], [36]]. Likewise, better health outcomes have been observed when efforts are made to train patients to effectively communicate with HCPs [37]. At best, direct patient communication, therefore, could improve MA and health outcomes. However, HCPs blaming or reprimanding patients for non-adherence can also be understood as direct patient communication, albeit with less favorable effects, as patients may feel judged in their decision-making. This may explain why eight in ten respondents reported direct patient communication as an available intervention, whereas only slightly more than one-third perceived it as successful. This discrepancy in communication can also be attributed to various factors previously described in the literature, including limited consultation time and workload, diverse skills in communication, health-literacy and language barriers [[38], [39], [40]]. Multimorbidity and polypharmacy may further complicate communication and adherence support [[41], [42], [43]]. Similarly, HCPs may find it challenging to provide clear and effective adherence support while managing multiple conditions and medications at the same time.

Technological solutions and collaborative care models reflect the increasing role of digital health and interdisciplinary collaboration in healthcare [44,45]. However, both were reported by fewer than a third of the respondents. Time constraints may hinder collaborative care [46], otherwise, infrastructure and technical issues, psychological barriers, and workload-related concerns have been identified as barriers to adopting digital health technologies by HCPs [47]. Experts reported heterogeneous adaptations during the COVID-19 pandemic, but these findings should be interpreted considering differences in national policies, healthcare disruption, and the retrospective nature of the responses. Some countries introduced telemedicine and facilitated access to medications, whereas respondents from other countries reported no specific adaptations. These findings highlight the importance of developing resilient and accessible MA support during major healthcare disruptions [48].

### 4.2 Implications for public health

These expert-reported findings identify interventions that may warrant further evaluation in MA programmes. Interpreting the response pattern through behavioural models helps explain why some interventions could be more effective than others. Direct communication with the patient and education mainly address the ‘perceptual’ side of adherence (beliefs about necessity, concerns) and motivation, which may help explain their widespread use [10]. In fact, closer follow-up and dose-dispensing/organizers could reduce practical barriers and improve patients' capabilities [8]. Technologically specific solutions (electronic prescriptions, reminders, telemedicine) also create opportunities and incentives, although uneven infrastructure across healthcare settings may limit their scope [7,25]. Additionally, collaborative approaches involving HCPs could be limited by professional boundaries, which may explain regional differences in their uptake. Hence, encouraging the creation of multidisciplinary groups for adherence management could be a more patient-tailored strategy potentially supporting more patient-centred care. Multidisciplinary interventions could include a short, structured adherence check at regular visits, documenting it in the electronic record, and monitoring specific indicators. This can help HCPs adopt proven measures and adapt them to the local context. Accordingly, the reported MA interventions should be evaluated in future implementation studies using objective measures of MA and patient outcomes.

### 4.3 Study strengths and limitations

A major strength of this study is its conduct within COST Action ENABLE a Europe-wide network of HCPs, patient representatives, researchers, and health-technology developers focused on MA [12,16,[48], [49], [50]]. The combination of a qualitative framework with a cross-sectional survey enabled the systematic analysis of expert-reported practices across 35 countries. However, the purposive, network-based recruitment strategy may have introduced selection bias, as participating experts may have been more involved in MA research, policy, or practice than other relevant professionals. The intended recruitment target was not reached in all countries, and both the number and professional composition of respondents varied across countries. The Eastern European sample included only 16 respondents from four countries and cannot represent the diversity of healthcare systems, policies, and MA interventions across the region. Regional analyses should therefore be considered exploratory and should not be generalised to entire regions or individual countries. Moreover, the study relied on expert reports and did not objectively assess national implementation or intervention effectiveness. COVID-19-related information was collected retrospectively and may have been affected by recall bias and respondents’ awareness of national or local initiatives. The self-administered survey did not allow researchers to probe individual responses and may have generated less detailed information than interviews or focus groups. Nevertheless, the focused open-ended questions enabled the systematic identification of recurring practices across a large multinational sample. Finally, although countries were grouped according to the Global Burden of Disease classification [13], substantial heterogeneity in healthcare systems, cultures, and economies remains within each region. Anyway, this classification facilitated comparative analysis by grouping countries with similar healthcare structures and socio-economic profiles.

### 4.4 Conclusion

Communication with patients and patient education were the medication adherence (MA) approaches most frequently reported by experts across Europe, and direct patient communication was the approach most often perceived as successful. Combining patient communication and education with behavioural, digital, and system-level approaches may warrant evaluation as a strategy for providing more comprehensive MA support. The descriptive map of adherence interventions in this study could drive future interventions to identify verifiable mechanisms to be considered priorities in future clinical and implementation studies.

## Ethical statement

The study was approved by the Research Ethics Committee of Malaga, Spain (Number 1932-on 29-04-2021), Croatia (Number 501-04 on 01-06-2021; Number 251-29-11-22-05 on 08-09-2022), the Republic of North Macedonia (Number 2005-133/3 on 06-05-2021), and Turkey (Number 24714 on 16-02-2022). In other countries, no formal approval was needed given the nature of the study according to local legislation. The study was conducted by the principles established in the Declaration of Helsinki, the Council of Europe Convention on Human Rights and Biomedicine, and the requirements established in each COST ENABLE country legislation. The study conformed to the norms of good clinical practice (art. 34 RD 223/2004; Community Directive 2001/20/CE) and the provisions of Regulation 2016/679 of the European Parliament and of the Council of April 27, 2016, on Data Protection (GDPR).

## Data availability

The data underlying this article are available in the article and in its online supplementary material. Additional data will be shared on reasonable request to the corresponding author.

## Author contributions

SM, JM, MK, EA, GH, MQ, IP, IT, ME, JB and FL developed the design for the two-part online survey. SM, EA and FL drafted the manuscript. All participated in the study design and reviewed the paper. All the authors have provided valuable contributions to the manuscript, read, and approved the final manuscript.

## Funding

This article is based upon work from 10.13039/501100000921COST Action “ENABLE”, CA19132, supported by 10.13039/501100000921COST (10.13039/501100000921European Cooperation in Science and Technology).

## Declaration of competing interest

The authors have nothing to declare. The authors declare that the research was conducted in the absence of any commercial or financial relationships that could be construed as a potential conflict of interest.
