# Supplementary material for: Mapping medication adherence interventions across Europe: Expert perspectives from 35 countries
Source: Public Health Pract (Oxf). 2026 Sep 9;12:100850. doi: 10.1016/j.puhip.2026.100850 (PMC13595210; doi:10.1016/j.puhip.2026.100850)
Supplement: Supplementary file 1 [file mmc2.docx]

Supplementary File 2

## 1.1 Supplementary Figures

## Figure S1. Frequencies of MA experts (n=140) by European regions.

**Notes:** Countries were classified according to the Global Burden of Disease regional framework used in the study (16).
**Western Europe:** Austria, Belgium, Cyprus, Denmark, Finland, France, Germany, Greece, Iceland, Ireland, Israel, Italy, Luxembourg, Malta, the Netherlands, Norway, Portugal, Spain, Sweden, Switzerland, and the United Kingdom.
**Central Europe:** Albania, Bosnia and Herzegovina, Bulgaria, Croatia, the Czech Republic, Hungary, Montenegro, North Macedonia, Poland, Romania, Serbia, Slovakia, and Slovenia.
**Eastern Europe:** Estonia, Latvia, Lithuania, Moldova, and Turkey.
No respondents were available from Denmark, Israel, Moldova, or the United Kingdom. Percentages shown in the figure refer to respondents, not to the number of countries.

## Figure S2. Countries of MA experts (n=140) by European regions.

**Figure S3.** Organisational affiliations reported by respondents, overall and by European region.

**Notes:** Total sample n=140 respondents. Values are n (% of respondents within the total sample or the corresponding region). Respondents could report more than one organisational affiliation.

## 1.2 Supplementary Tables

**Table S1.** Medication adherence interventions reported by experts, overall and by European region (question #5).

| **Intervention** | **Europe**  **N=140 (%)** | **Western**  **N=61 (%)** | **Central**  **N=63 (%)** | **Eastern**  **N=16 (%)** | **BH-adjusted exact p-value** | **Cramér’s V**  **(95% CI)** |
| --- | --- | --- | --- | --- | --- | --- |
| **Communication and Education** | **117 (83.6)** | **51 (83.6)** | **55 (87.3)** | **11 (68.8)** | **0.355** | **0.151 (0.000–0.300)** |
| Direct patient communication | 112 (80.0) | 49 (80.3) | 53 (84.1) | 10 (62.5) |  |  |
| Peer support | 9 (6.4) | 5 (8.2) | 2 (3.2) | 2 (12.5) |  |  |
| Education programmes | 7 (5.0) | 3 (4.9) | 2 (3.2) | 2 (12.5) |  |  |
| Shared decision-making | 5 (3.6) | 3 (4.9) | 2 (3.2) | - |  |  |
| Motivational interview | 3 (2.1) | 3 (4.9) | - | - |  |  |
| Longer consultations | 1 (0.7) | - | 1 (1.6) | - |  |  |
| **Follow-up and Monitoring** | **75 (53.6)** | **37 (60.7)** | **31 (49.2)** | **7 (43.8)** | **0.387** | **0.129 (0.000–0.276)** |
| Closer follow-up | 63 (45.0) | 31 (50.8) | 28 (44.4) | 4 (25.0) |  |  |
| Regimen interventions | 16 (11.4) | 9 (14.8) | 5 (7.9) | 2 (12.5) |  |  |
| Dose-dispensing | 8 (5.7) | 7 (11.5) | - | 1 (6.3) |  |  |
| Medication review | 5 (3.6) | 1 (1.6) | 3 (4.8) | 1 (6.3) |  |  |
| **Collaborative and Multidisciplinary Approaches** | **42 (30.0)** | **26 (42.6)** | **13 (20.6)** | **3 (18.8)** | **0.046** | **0.242 (0.041–0.396)** |
| Involvement of other HCPs | 24 (17.1) | 16 (26.2) | 7 (11.1) | 1 (6.3) |  |  |
| Caregiver involvement | 20 (14.3) | 11 (18.0) | 7 (11.1) | 2 (12.5) |  |  |
| Interventions on treatment burden | 2 (1.4) | 1 (1.6) | 1 (1.6) | - |  |  |
| **Technological Solutions** | **45 (32.1)** | **27 (44.3)** | **16 (25.4)** | **2 (12.5)** | **0.046** | **0.243 (0.042–0.397)** |
| Technological solutions specific for adherence | 41 (29.3) | 25 (41.0) | 14 (22.2) | 2 (12.5) |  |  |
| Medication organizers | 11 (7.9) | 6 (9.8) | 5 (7.9) | - |  |  |
| **Uncertain/Varied Approaches** | **23 (16.4)** | **11 (18.0)** | **11 (17.5)** | **1 (6.3)** | **0.650** | **0.099 (0.000–0.241)** |
| Other | 11 (7.9) | 5 (8.2) | 6 (9.5) | - |  |  |
| Unspecified adherence interventions | 10 (7.1) | 6 (9.8) | 4 (6.3) | - |  |  |
| Unaware | 2 (1.4) | - | 1 (1.6) | 1 (6.3) |  |  |
| Punishing measures | 1 (0.7) | - | 1 (1.6) | - |  |  |
| None | 20 (14.3) | 6 (9.8) | 8 (12.7) | 6 (37.5) |  |  |

**Abbreviations**: HCPs, healthcare professionals

**Notes**: Values are presented as n (% within the total sample or the corresponding region). Categories were quantified at respondent level. Each respondent was counted once within each macro-category but could contribute to more than one category. Regional differences in the proportion of respondents reporting each intervention category were explored using the Fisher–Freeman–Halton exact test. P-values were adjusted for the five macro-category comparisons using the Benjamini–Hochberg procedure. Cramér’s V with 95% confidence intervals is reported as a measure of the strength and precision of the association. Tests were omnibus and exploratory and did not account for clustering of respondents within countries. P-value less than 0.05 was considered statistically significant.

**Table S2**. Medication adherence interventions perceived as successful by experts, overall and by European region (question #6).

| **Interventions** | **Europe**  **N=140 (%)** | **Western**  **N=61 (%)** | **Central**  **N=63 (%)** | **Eastern**  **N=16 (%)** | **BH-adjusted exact p-value** | **Cramér’s V**  **(95% CI)** |
| --- | --- | --- | --- | --- | --- | --- |
| **Communication and Education** | **59 (42.1)** | **22 (36.1)** | **29 (46.0)** | **8 (50.0)** | **0.499** | **0.111 (0.000–0.255)** |
| Direct patient communication | 49 (35.0) | 17 (27.9) | 26 (41.3) | 6 (37.5) |  |  |
| Patient-HCP relationship | 6 (4.3) | 1 (1.6) | 4 (6.3) | 1 (6.3) |  |  |
| Education programmes | 5 (3.6) | 3 (4.9) | 1 (1.6) | 1 (6.3) |  |  |
| Motivational interview | 4 (2.9) | 4 (6.6) | - | - |  |  |
| Training of HCPs | 3 (2.1) | 2 (3.3) | - | 1 (6.3) |  |  |
| Peer support | 2 (1.4) | 1 (1.6) | 1 (1.6) | - |  |  |
| Longer consultations | 2 (1.4) | - | 1 (1.6) | 1 (6.3) |  |  |
| Shared decision-making | 2 (1.4) | 2 (3.3) | - | - |  |  |
| **Collaborative and Multidisciplinary Approaches** | **35 (25.0)** | **21 (34.4)** | **13 (20.6)** | **1 (6.3)** | **0.209** | **0.216 (0.000–0.369)** |
| Caregiver involvement | 12 (8.6) | 6 (9.8) | 6 (9.5) | - |  |  |
| Involvement of other HCPs | 12 (8.6) | 10 (16.4) | 2 (3.2) | - |  |  |
| Adherence programmes | 11 (7.9) | 4 (6.6) | 6 (9.5) | 1 (6.3) |  |  |
| Combined interventions | 3 (2.1) | 3 (4.9) | - | - |  |  |
| **Follow-up and Monitoring** | **28 (20.0)** | **12 (19.7)** | **15 (23.8)** | **1 (6.3)** | **0.499** | **0.133 (0.000–0.280)** |
| Closer follow-up | 14 (10.0) | 2 (3.3) | 11 (17.5) | 1 (6.3) |  |  |
| Regimen interventions | 5 (3.6) | 4 (6.6) | 1 (1.6) | - |  |  |
| Medication review | 5 (3.6) | 2 (3.3) | 3 (4.8) | - |  |  |
| Dose-dispensing | 4 (2.9) | 4 (6.6) | - | - |  |  |
| **Technological Solutions** | **18 (12.9)** | **9 (14.8)** | **7 (11.1)** | **2 (12.5)** | **0.878** | **0.051 (0.000–0.173)** |
| Specific technological solutions | 17 (12.1) | 9 (14.8) | 6 (9.5) | 2 (12.5) |  |  |
| Medication organizers | 1 (0.7) | - | 1 (1.6) | - |  |  |
| **Uncertain/Varied Approaches** | **21 (15.0)** | **13 (21.3)** | **6 (9.5)** | **2 (12.5)** | **0.499** | **0.157 (0.000–0.307)** |
| Individual approach | 11 (7.9) | 6 (9.8) | 4 (6.3) | 1 (6.3) |  |  |
| Other | 6 (4.3) | 3 (4.9) | 3 (4.8) | - |  |  |
| Unaware | 5 (3.6) | 4 (6.6) | - | 1 (6.3) |  |  |
| None | 22 (15.7) | 7 (11.5) | **12 (19.0)** | 3 (18.8) |  |  |

**Abbreviations**: HCPs, healthcare professionals

**Notes**: Values are presented as n (% within the total sample or the corresponding region). Categories were quantified at respondent level. Each respondent was counted once within each macro-category but could contribute to more than one category. Regional differences in the proportion of respondents identifying an intervention category as successful were explored using the Fisher–Freeman–Halton exact test. P-values were adjusted for the five macro-category comparisons using the Benjamini–Hochberg procedure. Cramér’s V with 95% confidence intervals is reported as a measure of the strength and precision of the association. Tests were omnibus and exploratory and did not account for clustering of respondents within countries. P-value less than 0.05 was considered statistically significant.

**Table S3.** Medication adherence initiatives reported by experts during the COVID-19 pandemic, overall and by European region (question #10).

| **Ad-hoc interventions** | **Europe**  **N=140 (%)** | **Western**  **N=61 (%)** | **Central**  **N=63 (%)** | **Eastern**  **N=16 (%)** |
| --- | --- | --- | --- | --- |
| **COVID-19 initiatives** |  |  |  |  |
| None | 57 (40.7) | 20 (32.8) | 29 (46.0) | 8 (50.0) |
| Unaware | 32 (22.9) | 21 (34.4) | 8 (12.7) | 3 (18.8) |
| Telemedicine | 22 (15.7) | 8 (13.1) | 12 (19.0) | 2 (12.5) |
| Easier access to medicines (e.g. via pharmacists, medicine home delivery) | 11 (7.9) | 6 (9.8) | 3 (4.8) | 2 (12.5) |
| Other | 11 (7.9) | 6 (9.8) | 4 (6.3) | 1 (6.3) |
| Informative campaigns, lectures, webinars | 9 (6.4) | 3 (4.9) | 5 (7.9) | 1 (6.3) |
| Actions related to COVID vaccination | 4 (2.9) | 2 (3.3) | 2 (3.2) | - |
| Unspecified national actions | 3 (2.1) | - | 3 (4.8) | - |
| Education of HCPs | 2 (1.4) | - | 2 (3.2) | - |
| Development of special guidelines | 1 (0.7) | - | 1 (1.6) | - |

**Abbreviations**: HCPs: healthcare professionals

**Notes**: Values are presented as n (% within the total sample or the corresponding region). Categories were quantified at respondent level. Each respondent was counted once within each category but could report more than one COVID-19-related initiative. Findings are descriptive and reflect experts’ retrospective reports of initiatives implemented during the COVID-19 pandemic. Inferential regional comparisons were not performed because the categories were non-mutually exclusive and several categories had sparse counts, precluding a meaningful overall comparison across regions.
