# Supplementary material for: Mapping medication adherence interventions across Europe: Expert perspectives from 35 countries
Source: Public Health Pract (Oxf). 2026 Sep 9;12:100850. doi: 10.1016/j.puhip.2026.100850 (PMC13595210; doi:10.1016/j.puhip.2026.100850)
Supplement: Supplementary file 2 [file mmc3.docx]

Supplementary File 3

**Table S1.** Coding of responses to question #1: Which professions are involved in medication adherence in your country and what is their role in evaluating, monitoring, documenting and/or improving adherence?

| **Codes** | **Examples of quotes** |
| --- | --- |
| Physicians: primary care, family doctors (GPs) and medical specialists, clinical pharmacologist | *“Physician has the overall responsibility of medication treatment.”*  *“Mainly doctors…”* |
| Pharmacists (community and hospital, clinical pharmacists) | *“…pharmacists (still very limited!)…”*  *“The professions involved in this [area] are pharmacists…”* |
| Nurses | *“Nurse: all roles”* |
| Dentists | *”Dental Professionals: monitor, document and improve.”* |
| Home care services | *”…home nurses.”* |
| Psychologists | *“…and psychologists deal with drug adherence.”* |
| Other (e.g. medical technicians, physiotherapists, midwives, assistants, social services) | *“…nursing aides.”*  *“…medical technicians”*  *“…occupational therapists in more institutionalized settings.”* |
| No professions involved | *“There are no professionals involved…”* |
| National health insurance institutes | *“Beside them institution such as National Healthcare insurance fund evaluate medication adherence.”* |
| Researchers | *“researchers (mainly during clinical trials)”* |

**Table S2.** Coding of responses to question #5: If a patient is identified as being non-adherent, what interventions can health professionals offer to improve adherence (for example, give consultation, organize closer follow-up, refer to an adherence intervention/program, mobile applications)?

| **Categories & codes** | **Example quotes** |
| --- | --- |
| **Communication and education** | |
| Direct patient communication (and relevant/appropriate education) | *“Mainly, to assess through a clinical interview the obstacles that are motivating non-adherence and to seek solutions to it.”*  *“They can only be consulted during a general visit.”* |
| Peer support (incl. collaboration with patient associations) | *“referral to peer-support groups, patient empowerment societies…”*  *“...participation in patient advocacy/cancer survival groups.”* |
| Education programmes (incl. self-care training) | *“Self-care training programmes”*  *“…for some special diseases can send for specialized lessons.”* |
| Shared decision-making | *“…patient involvement in decision-making on treatment”* |
| Motivational interview | *“…perform motivational interviewing…”* |
| Longer consultations | *“…longer consultations.”* |
| **Follow-up and monitoring** | |
| Closer follow-up | *“more frequent visits to the doctor”* |
| Regimen interventions | *“Medication could also be modified (e.g. change dosage form of the medication, change to extended-release products, simplify patient's treatment).”* |
| Dose-dispensing | *“…pharmacy dispensed doses...”* |
| Medication review | *“…perform MUR”* |
| **Technological solutions** | |
| Technological solutions specific for adherence (e.g. mobile apps, reminders) | *“...installing applications on mobile devices that will inform the patient not to miss the time to take the medicine ...”*  *“…use of mobile or other applications…”* |
| Medication organizers | *“Use of pill organizers through pharmacies”* |
| **Collaborative and multidisciplinary approaches** | |
| Involvement of other HCPs (incl. home care) | *“Collaboration with GP and homecare staff”* |
| Caregiver involvement (incl. education) | *“...involve someone from the patient's family to help...”* |
| Interventions on treatment burden | *“…if the patient does not have sufficient financial resources, contact the Social Worker ...”* |
| **Uncertain/varied approaches** | |
| Other | *“develop a medication plan appropriate to their needs and beliefs”* |
| Unspecified adherence interventions | *“…be invited to participate in dedicated programs…”* |
| Unaware | *“I don't know about programs”* |
| Punishing measures | *“…the physicians can refuse his further examinations...”* |
| **No methods available** | *“…there are no systematic adherence programs on national level....”* |

**Abbreviations**: HCPs, healthcare professionals; MUR, Medicines Use Review

**Table S3.** Coding of responses to question #6: What would you identify as the most successful adherence interventions in your country within the interventions you mentioned in your answer to question 5?

| **Categories & codes** | **Example quotes** |
| --- | --- |
| **Communication and education** | |
| Direct patient communication (incl. education) | *“The [explanation] to the patient and the patient understanding of seriousness of his/her disease and why it is so important to adhere the treatment.”* |
| Patient-HCP relationship | *“A closer doctor-patient relationship based on mutual trust.”* |
| Education programmes (incl. self-care training) | *“[Self-care] training programmes”* |
| Motivational interview | *“…motivational interviewing for unintentional [nonadherence]”* |
| Training of HCPs | *“For the professionals, their training.”* |
| Peer support (incl. patient associations) | *“Training with expert [patients]”* |
| Longer consultations | *“...consultation must last long…”* |
| Shared decision-making | *“…(HCPs listen to the patient and seeking solutions together)…”* |
| **Collaborative and multidisciplinary approaches** |  |
| Caregiver involvement (incl. education) | *“cooperation with family caregiver”* |
| Involvement of other HCPs (incl. home care) | *“…being able to involve other actors such as pharmacologists and pharmacists is also very important to improve adherence ...”* |
| Adherence programs / interventions | *“Development and implementation of organized PSP (patients supporting programmes)…”* |
| Combined interventions | *“In my experience, a combination of interventions is often the best one…”* |
| **Follow-up and monitoring** |  |
| Closer follow-up | *“...provide additional consultation or provide closer follow-up”* |
| Regimen interventions | *“Simplification of treatments”* |
| Medication review | *“Medication use review by pharmacists…”* |
| Dose-dispensing | *“Pharmacy dispensed doses”* |
| **Technological solutions** |  |
| Technological solutions specific for adherence | *“...using reminder tools and mobile applications…”* |
| Medication organizers | *“Pillboxes…”* |
| **Uncertain/varied approaches** |  |
| Individual approach | *“...depends on the individual needs of the patients.”* |
| Other | *“…medical call center service / online consultations”* |
| Unaware | *“It is difficult to say how effective, in my opinion, any educational campaigns are…”* |
| **None** | *“There is no best practice for that. There is a clear unmet medical need on this field”* |

**Abbreviations**: HCPs, healthcare professionals

**Table S4.** Coding of responses to question #10: Are there any specific initiatives or actions for improving adherence during COVID pandemic? If yes, please specify them.

| **Codes** | **Example quotes** |
| --- | --- |
| Informative campaigns, lectures, webinars | *“Information through the media and initiatives of medical associations”* |
| Unspecified national actions | *“During the COVID pandemic, were only a few separate attempts (activities) linked to the medication adherence organized by Doctors of the primary care in coordination with the Center for Public Health.”* |
| Development of special guidelines | *“Development of special guidelines by medical experts from our country”* |
| Telemedicine (incl. e-prescription) | *“The use of telemedicine, the electronic recipe for patients to avoid delays…”* |
| Easier access to medicines (e.g. via pharmacists, medicine home delivery) | *“…there was a possibility for patients to get a drug from a pharmacy without prescription for chronic disease treatment…”* |
| Education of HCPs | *“…a series of webinars focused on medication adherence for pharmacists”* |
| Other | *“More patients are included to outpatient nursing services like assistance at home.”* |
| Actions related to COVID vaccination | *“There were actions to offer advantages to COVID-vaccinated people”* |
| Unaware | *“I suppose that no.”* |
| None | *“There are no specific actions.”* |

**Abbreviations**: HCPs, healthcare professionals
