# Supplementary material for: Mapping medication adherence interventions across Europe: Expert perspectives from 35 countries
Source: Public Health Pract (Oxf). 2026 Sep 9;12:100850. doi: 10.1016/j.puhip.2026.100850 (PMC13595210; doi:10.1016/j.puhip.2026.100850)
Supplement: Supplementary file 3 [file mmc1.doc]

Supplementary file 1

# Pilot study results

**Table S1**. Characteristics of the respondents (n=12) in the pilot study.

|  | **n** | **Percent** |
| --- | --- | --- |
| **Informed consent** |  |  |
| Agreed | 12 | 100% |
| **Country** |  |  |
| Bulgaria | 3 | 25% |
| Croatia | 3 | 25% |
| Spain | 2 | 16.7% |
| Sweden | 2 | 16.7% |
| Turkey | 2 | 16.7% |
| **Organization** |  |  |
| Research/academia | 4 | 33.3% |
| Hospital | 3 | 25 % |
| Professional association | 3 | 25 % |
| Community pharmacy | 1 | 8.3% |
| Governmental | 1 | 8.3% |
| Other | 1 | 8.3% |

**Table S2.** Coding of responses to question #1: Which professions are involved in medication adherence in your country and what is their role in evaluating, monitoring, reporting and/or improving adherence?

| **Codes** | **Examples of quotes** |
| --- | --- |
| Physicians/Doctors: Primary Care and Medical Specialists | *“mainly primary care physicians (general practitioners) and medical specialist”* |
| Pharmacists | *“Pharmacists with paramount role in drug use and adherence monitoring in case of [prescription-only medicines]…”* |
| Nurses | *“The nurses [control] lab samples and if the patients is on medication that needs to be monitored.”* |
| Dentist | *“Medical doctors and dentists as the only legitimate prescribers of [prescription-only medicines]…”* |
| Home care services | *“…but also people who [work] in home care services who [work] near the patients.”* |
| Prescribers | *“…and prescribers are involved in this process.”* |

**Table S3. Coding of responses to question #2: How can healthcare professionals evaluate patients' medication adherence in your country?**

| **Codes** | **Examples of quotes** |
| --- | --- |
| Patients’ self-report | *“In general by direct questioning of the patient.”* |
| Caregiver’s report | *“…reports from primary caregivers (typically relatives)…”* |
| Dispensing information | *“…some information on dispensing patterns and medication adherence…”* |
| Clinical record information | *“…extracting information from EHR”* |
| Direct methods of diagnosing treatment adherence | *“…measurement of drug concentration in blood/urine…”* |
| HCP’s evaluation (e.g. based on clinical signs) | *“Evaluation of clinical presentations…”* |
| Specific experiences/situations | *“Some pharmacies are preparing weekly medications packs for patients…”* |
| No methods available | *“We have no standard procedures on evaluating medication adherence.”* |
| Do not know | *“I do not know”* |

**Table S4.** Coding of responses to question #3: What kind of patient level data on medication adherence healthcare professionals have access to?

| **Codes** | **Examples of quotes** |
| --- | --- |
| Dispensation data | *“…can check whether patients take medication or not from the pharmacy”* |
| Prescriptions | *“Their data is limited to dynamics on prescribing medicines.”* |
| Clinical records | *“…only what is documented in medical records”* |
| Dose-recording technology | *“…insulin pump users where there is traceability of insulin administration.”* |
| Patients’ self-report | *“…direct feedback interview with the patient…”* |
| No access to patient-level data | *“…difficult to get data on individual level.”* |
| Pharmacy data (unspecified) | *“You can request from the hospital pharmacy data collected in an out-of-hospital pharmacy…”* |

**Tables S5.** Coding of responses to question #4: If a patient is identified as being non-adherent, what options and tools can health professionals provide?

| **Codes** | **Examples of quotes** |
| --- | --- |
| Direct patient communication | *“…direct talk and explanation why medication is important”* |
| Medication organizer | *“Pill organizer…”* |
| Dose-dispensing | *“…pre-packed bags with tablets to take…”* |
| Regimen interventions | *“…ensure a proper drug regimen for each patient”* |
| Electronic tools (e.g. mobile apps, including reminders) | *“…different types of reminder-services (Apps)”* |
| Closer follow-up | *“Intensify clinical follow-up…”* |
| Collaboration between HCPs | *“…could contact the prescribing physician”* |
| Caregiver education | *“…and education of … the caregiver about the illness, the drug action and side effects, dosing, etc…”* |
| Controlled dispensing | *“…patient needs to get to the hospital/PHCC in order to receive the medicine”* |
| No methods available | *“Nothing standardized”* |

**Tables S6.** Coding of responses to question #5: If a healthcare professional identifies a patient to be non-adherent, where can it be reported or documented?

| **Codes** | **Examples of quotes** |
| --- | --- |
| Patient’s medical records | *“non-adherence can be documented in the medical records (hospital or ambulatory)”* |
| Dispensing program | *“…and (if the patient agrees) noted in the personal file within the dispensing computer program.”* |
| Inform the prescriber | *“...report only to the physician who manages the treatment”* |
| Not documented | *“The pharmacists working at the pharmacy have no possibility to document this on an individual level…”* |
| Do not know | *“Not sure about this”* |

**Table S7.** Coding of responses to question #6: Describe the existing national policies, guidelines, frameworks, or campaigns on medication adherence in your country.

| **Codes** | **Examples of quotes** |
| --- | --- |
| National campaigns | *“…provide public campaigns on the importance of medication adherence.”* |
| National strategies | *“We have [documented] the problem in our national strategies…”* |
| National guidelines | *“…within the framework of the provisions of the "Good Pharmacy Practices Guide" and the "Regulation on Pharmacies and Pharmacists"…”* |
| National projects | *“…some initiatives by the marketing authorization holders.”* |
| None | *“No such exist”* |
| Don’t know | *“I do not know…”* |

**Table S8.** Coding of responses to question #7: What would you identify as the best practices and the most successful adherence management strategies in your country?

| **Codes** | **Examples of quotes** |
| --- | --- |
| Shared decision making | *“Shared [decision] making interventions…”* |
| Direct patient communication | *“Regular patient consultation by pharmacist and prescriber…”* |
| Guidelines | *“to follow the clinical practice [standards]”* |
| Training of HCPs | *“The awareness and training of healthcare professionals.”* |
| Information on patient’s adherence | *“Adequate identification in the clinical history of the information on adherence to treatment…”* |
| Technological solutions | *“Mobile applications that give the patient individualized support…”* |
| Dose-dispensing | *“…preparation of individualized weekly medication packs.”* |
| Not available | *“Unfortunately we do not have any [practices] that are publicly [available]…”* |
| Do not know | *“…I do not have the overview of the situation”* |

**Table S9. Coding of responses to question #8: What kind of planned institutional, governmental, or non-governmental initiatives on medication adherence exist in your country?**

| **Codes** | **Examples of quotes** |
| --- | --- |
| National/official campaigns are planned | *“…managing an operation on pharmaceutical services at open-care pharmacies with the objective of improving medication adherence.”* |
| None | *“I would say there are no planned public [initiatives] on medication adherence”* |
| Do not know | *“I [don’t] know for such initiatives.”* |

**Table S10. Coding of responses to question #9: How do you evaluate implementation and adequacy of implementation of medication adherence initiatives in your country?**

| **Codes** | **Examples of quotes** |
| --- | --- |
| Evaluation exists but not completely developed | *“…surveys before and after conducted public campaigns indicate…”* |
| No evaluation procedure | *“We do not evaluate implementation [on] medication initiatives on adherence.”* |
| Do not know/Not aware | *“I actually do not know.”* |

**Table S11.** Coding of responses to question #10: What would you identify as an unmet need regarding medication adherence in your country?

| **Codes** | **Examples of quotes** |
| --- | --- |
| Lack of national standards | *“Official and standardized guidelines.”* |
| Lack of data on patient’s adherence | *“An easy way to follow your patient on an individual level”* |
| Need for technological solutions | *“Need for alarms in clinical records”* |
| Lack of monitoring of adherence | *“…more frequent controls regarding medication adherence”* |
| Lack of collaboration between HCPs | *“Need for coordination among different levels of care in the health systems”* |
| Need for training | *“Need for training from university”* |
| Adherence not seen important | *“no specific focus of medical specialist on medication adherence”* |
| Need for more research | *“only sporadic data on medical adherence coming in the form of single PhD studies for example”* |
| No tools to improve adherence | *“…lack of …tools and motivating practices.”* |

**Table S12.** Coding of responses to question #11: What barriers exist for implementing medication adherence policy in your country (e.g., lack of resources, financing, activities, support)?

| **Codes** | **Examples of quotes** |
| --- | --- |
| Lack of awareness | *“We believe that the biggest [barrier] is lack of [awareness] of this problem.”* |
| Not seen important | *“…not many are aware of its importance.”* |
| Politics and lack of legislation | *“The lack of a legal infrastructure is an obstacle.”* |
| Need for training | *“Lack of ...training in this field for health professionals.”* |
| Lack in IT | *“Lack of good computer systems…”* |
| Lack of incentives for adherence management | *“probably lack of measures to [evaluate] potential outcomes and benefits, leading to lack of initiative to spare resources”* |
| Lack of resources | *“The main barriers are the lack of resources and activities…”* |
| Lack of collaboration between HCPs | *“…very important that [barriers]…between professions (doctors, nurses and pharmacists) hamper the development of efforts…”* |
| Lack of patient-level information | *“…the lack of …collecting relevant information from each patient.”* |
| Low health literacy | *“The health literacy rates of our patients are not at a sufficient level.”* |
| Lack of research funding | *“Lack of funding for research in this area.”* |

**Table S13.** Coding of responses to question #12: How did COVID affect management of adherence and problems regarding medication adherence?

| **Codes** | **Examples of quotes** |
| --- | --- |
| Negative effects of COVID-19 on medication adherence | *“Negatively in terms that the patients could not visit their GPs, miss prescriptions or advise.”* |
| Positive effects of COVID-19 on medication adherence | *“The Covid-19 pandemic raised awareness in terms of being a critical period in which the importance of patients' medication adherence was better understood…”* |
| Initiatives to stop negative impact of pandemic | *"Pharmacists are following standard procedures and maintaining patient education and consultations…"* |
| Don’t know | *“I am not sure”* |

**Table S14.** Final structure of the questionnaire and analytical approach used in the present study.

| Domain | Question | Final survey question | Analytical approach |
| --- | --- | --- | --- |
| Professional roles in medication adherence management | 1 | **Which professions are involved in medication adherence in your country, and what is their role in evaluating, monitoring, documenting, and/or improving adherence?** | Framework-based coding of professional groups, followed by respondent-level calculation of frequencies and percentages. |
| Assessment of medication adherence | 2 | How can healthcare professionals evaluate patients’ medication adherence in your country? | Not included in the present analysis. |
| Availability of adherence data | 3 | What kind of patient-level data on medication adherence do healthcare professionals have access to? | Not included in the present analysis. |
| Documentation of non-adherence | 4 | If a healthcare professional identifies a patient as non-adherent, where can this be documented? | Not included in the present analysis. |
| Medication adherence interventions | 5 | **If a patient is identified as non-adherent, what interventions can healthcare professionals offer to improve adherence (e.g. consultation, closer follow-up, referral to an adherence intervention or programme, or mobile applications)?** | Framework-based coding into categories and subcategories, followed by respondent-level calculation of overall and region-specific frequencies and percentages. |
| Perceived success of medication adherence interventions | 6 | **Among the interventions mentioned in response to Question 5, which would you identify as the most successful medication adherence interventions in your country?** | Framework-based coding into categories and subcategories, followed by respondent-level calculation of overall and region-specific frequencies and percentages. |
| National policies and initiatives | 7 | Describe the existing and planned national policies, guidelines, frameworks, or campaigns on medication adherence in your country. If relevant documents are available, please attach them or provide an internet address. | Not included in the present analysis. |
| Unmet needs | 8 | What would you identify as an unmet need regarding medication adherence in your country? | Not included in the present analysis. |
| Barriers to medication adherence management | 9 | What barriers to medication adherence management exist in your country (e.g. lack of resources, funding, activities, or institutional support)? | Not included in the present analysis. |
| Medication adherence initiatives during the COVID-19 pandemic | 10 | **Were any specific initiatives or actions introduced to improve medication adherence during the COVID-19 pandemic? If yes, please describe them.** | Framework-based coding of reported initiatives, followed by respondent-level calculation of overall and region-specific frequencies and percentages. |

**Notes:** Questions included in the present analysis are shown in bold.
